# Prostate Cancer Nodal Staging: Using Deep Learning to Predict ^68^Ga-PSMA-Positivity from CT Imaging Alone

**DOI:** 10.1038/s41598-020-60311-z

**Published:** 2020-02-25

**Authors:** A. Hartenstein, F. Lübbe, A. D. J. Baur, M. M. Rudolph, C. Furth, W. Brenner, H. Amthauer, B. Hamm, M. Makowski, T. Penzkofer

**Affiliations:** 1 Charité – Universitätsmedizin Berlin, corporate member of Freie Universität Berlin, Humboldt-Universität zu Berlin, Department of Radiology, Augustenburger Platz 1, 13353 Berlin, Germany; 2 Charité – Universitätsmedizin Berlin, corporate member of Freie Universität Berlin, Humboldt-Universität zu Berlin, Department of Nuclear Medicine, Charitéplatz 1, 13353 Berlin, Germany; 3 Berlin Institute of Health (BIH), Anna-Louisa-Karsch-Str. 2, 10178 Berlin, Germany; 4 Institute for Diagnostic and Interventional Radiology, Klinikum rechts der Isar der Technischen Universität München, Ismaninger Straße 22, D-81675 München, Germany

**Keywords:** Diagnostic markers, Cancer imaging

## Abstract

Lymphatic spread determines treatment decisions in prostate cancer (PCa) patients. 68Ga-PSMA-PET/CT can be performed, although cost remains high and availability is limited. Therefore, computed tomography (CT) continues to be the most used modality for PCa staging. We assessed if convolutional neural networks (CNNs) can be trained to determine 68Ga-PSMA-PET/CT-lymph node status from CT alone. In 549 patients with 68Ga-PSMA PET/CT imaging, 2616 lymph nodes were segmented. Using PET as a reference standard, three CNNs were trained. Training sets balanced for infiltration status, lymph node location and additionally, masked images, were used for training. CNNs were evaluated using a separate test set and performance was compared to radiologists’ assessments and random forest classifiers. Heatmaps maps were used to identify the performance determining image regions. The CNNs performed with an Area-Under-the-Curve of 0.95 (status balanced) and 0.86 (location balanced, masked), compared to an AUC of 0.81 of experienced radiologists. Interestingly, CNNs used anatomical surroundings to increase their performance, “learning” the infiltration probabilities of anatomical locations. In conclusion, CNNs have the potential to build a well performing CT-based biomarker for lymph node metastases in PCa, with different types of class balancing strongly affecting CNN performance.

## Introduction

Prostate cancer (PCa) is the most common malignant cancer in men worldwide, and the second most common cause of cancer related death in men^1^. Patients with intermediate or high-risk PCa undergo regular staging examinations in order to determine if the tumor has spread beyond the prostate. As treatment success is highly dependent on the presence of systemic spread^2,3^, staging procedures with high sensitivity and specificity are necessary.

Standard of care imaging for PCa staging typically includes contrast-enhanced computed tomography (CT) and Technetium-99m-methylene diphosphonate bone scans^4,5^. Despite the continued recommendation of CT in staging, it has been shown that predicting lymph node infiltration (LNI) with CT scans is not very reliable^6,7^, with one study reporting a sensitivity and specificity of only 42% and 82%^8^. This low performance is most likely due to the limited morphological criteria used to define a lymph node as positive for infiltration, with size being the most relevant^9^. A threshold of 8–10 mm is often used despite the fact that 80% of lymph node metastases are less than 8 mm in the short axis^10^. Further criteria, such as status of hilum fat, nodal shape, and enhancement characteristics are used to aid diagnosis, but it remains difficult to exclude LNI in large benign hyperplastic nodes or detect it in small nodes below the size threshold^11^.

In 2012 imaging agents binding to Prostate Specific Membrane Antigen (PSMA) were introduced, leading to the development of PSMA PET/CT^8^. PSMA, an integral membrane glycoprotein expressed 100–1000 fold on membranes of PCa cells compared to prostate cells, has been shown to correlate with aggressive disease, disease recurrence, and metastasis^12–14^, and radio-tracer targeting of PSMA in conjunction with CT has been shown in a systematic review and meta-analysis of 5 studies to predict LNI with a sensitivity and specificity of 80% and 97% respectively^15^. PSMA PET/CT has been used to detect PCa in the prostate, soft tissue, and bone, and has been shown to detect LNI in nodes even under 10 mm in size, with one study reporting a 60% detection rate for nodes between 2–5 mm^16,17^.

Even though PSMA PET/CT has proven to be very valuable in PCa staging, it remains of limited availability and hybrid imaging such as PET/CT is associated with high costs. The goal of this study was to evaluate – using 68Ga-PSMA PET/CT as a reference standard – if it is possible to elucidate the status of lymph nodes based on contrast-enhanced CT images alone using deep learning in the form of convolutional neural networks (CNNs).

## Materials and Methods

### Imaging datasets

Inclusion criteria for this retrospective study was the availability of a 68Ga-PSMA PET/CT examination with parallel contrast-enhanced CT examination performed between September 2013 and April 2017. All patients had histopathologically verified prostate cancer that warranted staging examinations. Exclusion criteria were non-contrast or low-dose only CT examination, insufficient image quality, and follow-up studies (only the first 68Ga-PSMA PET/CT of each patient was included). Of 738 patients, 549 patients (68.7 ± 7.54 [45–87] years, PSA 20.9 ± 94.6 [0–1423] ng/ml) fulfilled our inclusion criteria. The study was approved by the Charité Ethics Committee, and due to the retrospective design, the need for informed written consent was waived by the same review board, in accordance with institutional guidelines and regulations. The study was performed in accordance with the Declaration of Helsinki.

All patients had received 68Ga-PSMA PET/CT examinations for clinical purposes during the course of treatment. A standard 68Ge/68Ga generator (Eckert and Ziegler Radiopharma GmbH, Berlin, Germany) was used for 68Ga production, and PSMA- HBED-CC (ABX GmbH, Radeberg, Germany) labelling with 68Ga was performed according to the previously described method^18^. All PET/CT images were acquired using a Gemini Astonish TF 16 PET/CT scanner (Phillips Medical Systems, Best, The Netherlands) after intravenous injection of 68Ga- PSMA-HBED-CC^19^ using 3-D acquisition mode for all PET scans.

**Figure 1:**
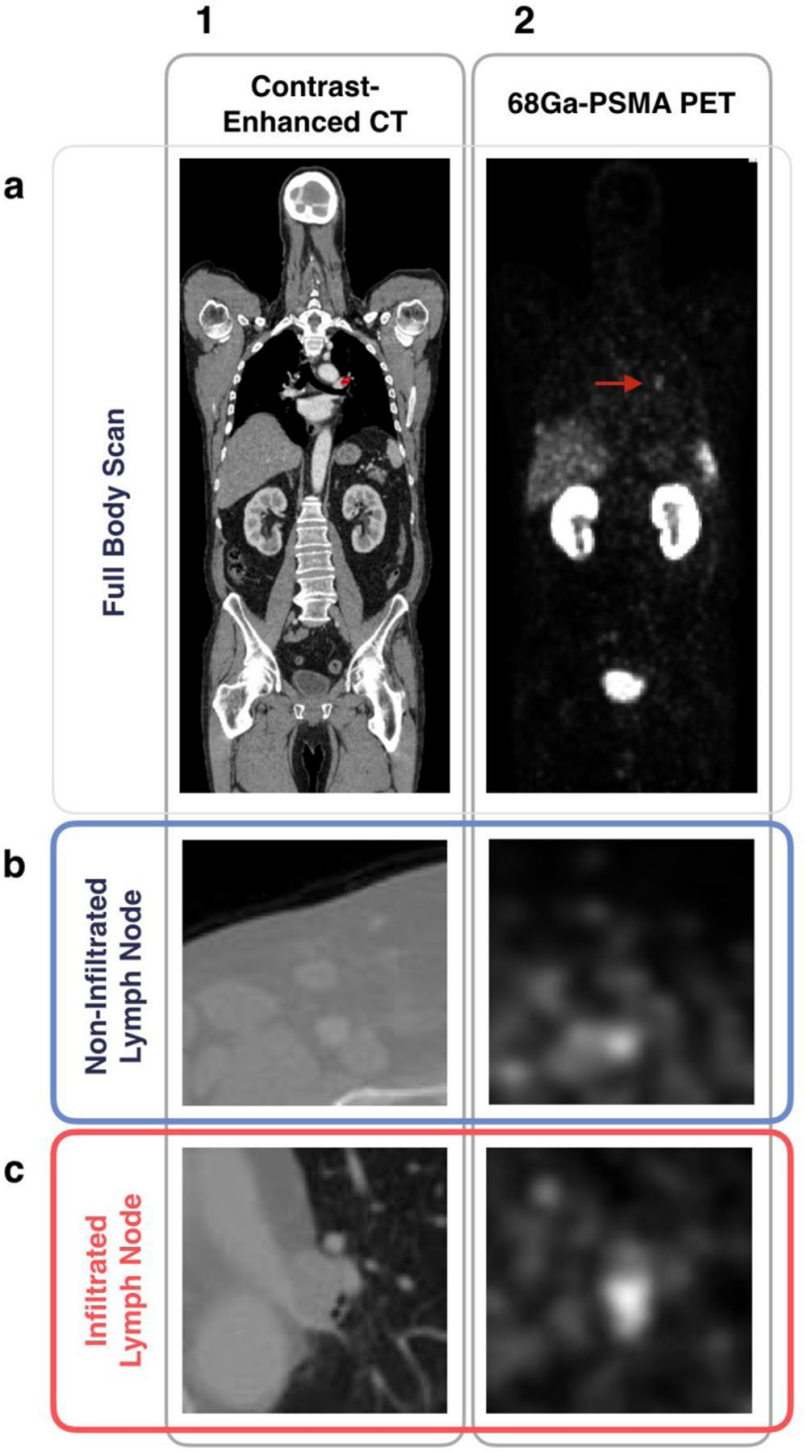
Generation of Labelled Dataset. (**a**) Imaging of a single patient with (1) a contrast-enhanced CT scan and (2) a 68Ga-PSMA PET scan. An average of 4.72 ± 0.77 lymph nodes were selected and semi-automatically segmented for each patient. A single lymph node positive for infiltration by PCa can be seen in the mediastinal region outlined in red in the CT image in (a1), and demarcated by a red arrow in PET scan in (a2). Using the 68Ga-PSMA PET/CT as our reference standard, a label for infiltration status by prostate cancer (either positive or negative) was assigned on a per lymph node basis. (**b**) An example of a negative 68Ga-PSMA PET/CT image pair in which the centered lymph node does not exceed background. (**c**) An example of a positive image pair.

Semi-automated manual three dimensional segmentation of lymph nodes was performed using the MITK software suite (MITK v. 2016.3.0, DKFZ, Heidelberg, Germany)^20^. Using the PSMA PET image as ground truth, a label of positive or negative for tumor infiltration was generated for each lymph node in consensus of two radiologists experienced in hybrid imaging, correlated with SUVmax. Figure 1 shows an example of a 68Ga-PSMA PET/CT full body scan and two selected lymph nodes, one positive and one negative for infiltration. In addition to the tumor infiltration label, the position of each lymph node in the body was manually assigned a categorical variable from a set of 9 possible categories (inguinal, iliacal (including obturator fossa), perirectal, (ascending) retroperitoneal, axillary, mediastinal, supra or infraclavicular, and cervical).

### Patient collective and dataset generation

**Figure 2:**
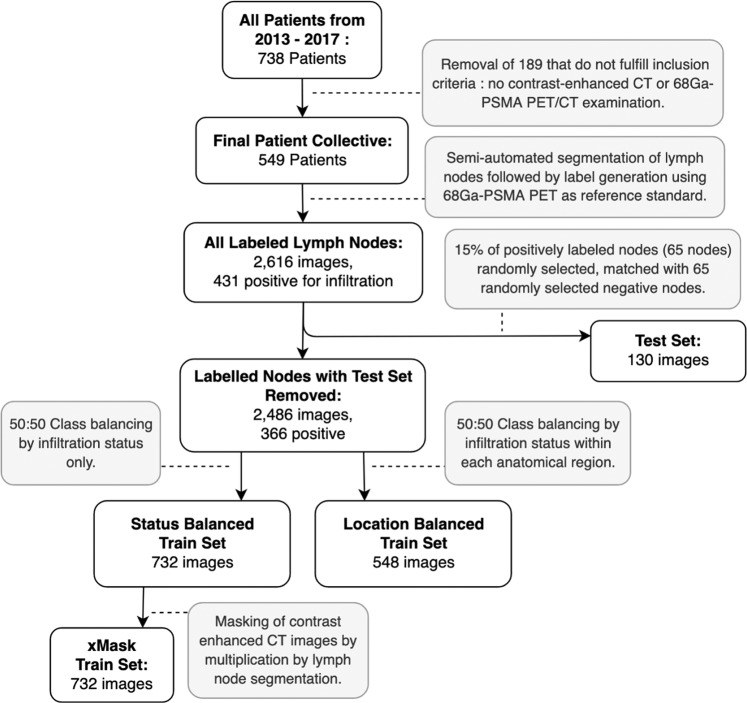
Dataset Generation Flowchart. Diagram describing generation of train and test datasets. Three train datasets shown, (status balanced, location balanced and xMask) were used to train three distinct neural networks. All neural networks and experts were tested and compared using a separate test set of 130 images, which was withheld from the neural networks during training. 50:50 class balancing was performed by taking all available infiltrated lymph nodes and randomly selecting an equally sized set of non-infiltrated lymph nodes, either from all available non-infiltrated nodes or from nodes within the same location category.

**Figure 3:**
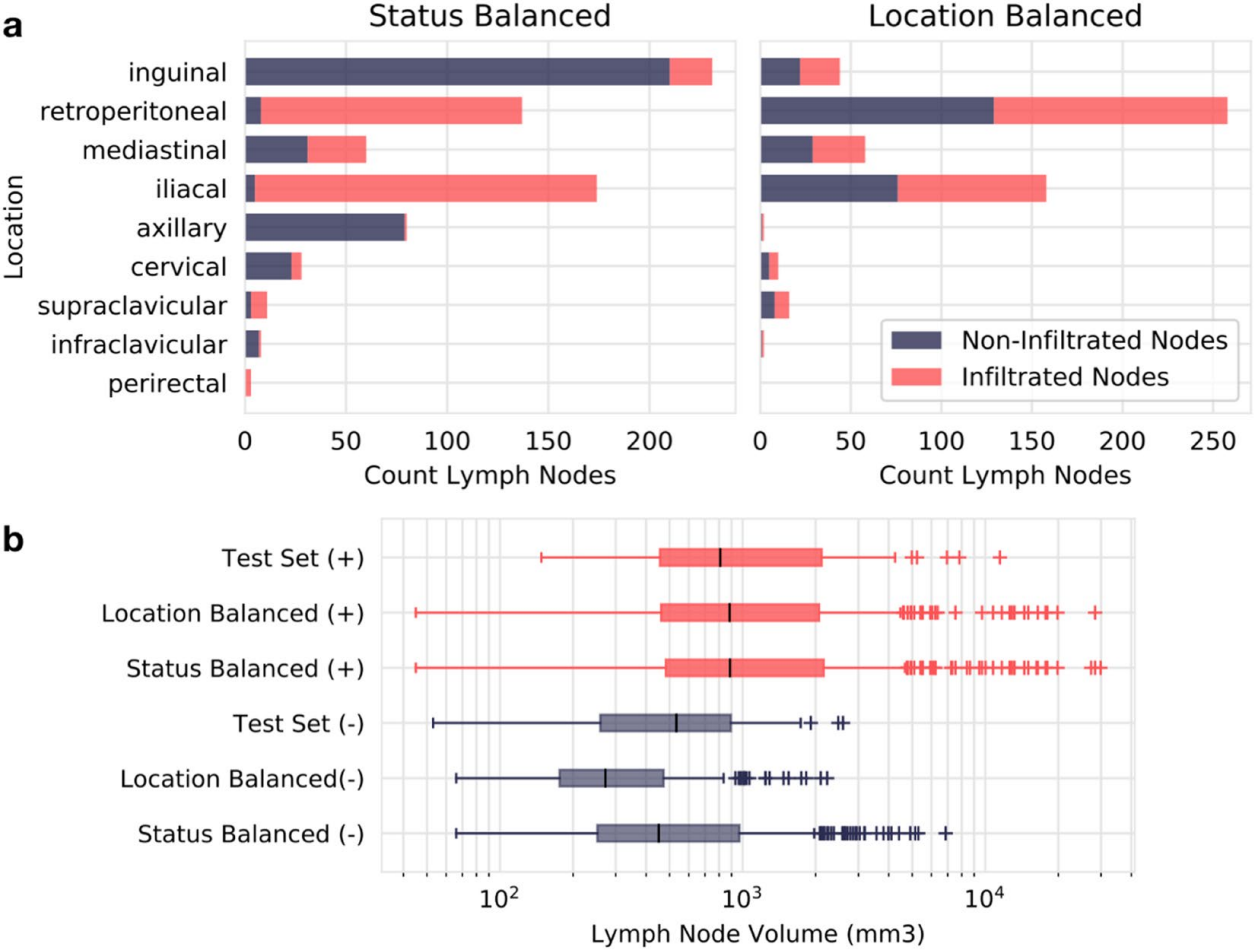
Dataset Regional and Volume Distributions. (**a**) The final distribution of lymph node images by location and infiltration status for the two training sets, referred to as ‘status balanced’ with 732 images and ‘location balanced’ with 548 images. (**b**) Boxplots depicting volume distribution for the location and status balanced training sets and test set grouped by infiltration status. Due to considerable overlap of the two distributions, size or volume is not a powerful indicator of infiltration.

A final set of 549 patients fulfilled the inclusion criteria. An average of 4.72 ± 0.77 (SD) lymph nodes were segmented and labelled in each patient resulting in a total of 2,616 labelled lymph nodes, with 431 of these labelled as positive for infiltration. Figure 2 shows how these images were used to generate test and training datasets, and is explained as follows. A set of 130 lymph nodes was set aside for testing all CNNs and experts. This test set was created by taking 15% of the available positive nodes (65 nodes) and matching with 65 randomly selected negative nodes to create a 50:50 class balanced set. The remaining 366 positive nodes were matched with 366 randomly selected negative nodes to create a 50:50 class balanced set referred to as the ‘status balanced’ training set, with a total of 732 lymph nodes. The majority of lymph nodes in the status balanced and test dataset were in the inguinal region (32%), followed by the iliacal region (23%), and retroperitoneal region (19%). Figure 3a shows anatomical distribution by training set. To investigate effects of anatomical localization on classification results, the same 366 positive nodes used to create the status balanced set were sorted by anatomical category and matched to randomly selected negative nodes from within the same anatomical category, thus creating a 50:50 class balanced set with 548 lymph nodes, referred to as the ‘location balanced’ training set.

### Neural network training

Images were resampled to an isotropic resolution of 1 × 1 × 1 mm^3^. A volume of 80 × 80 × 80 mm^3^ was cropped around the lymph node, centered at the center point of the manual lymph node segmentation. Image augmentation was performed online during model training, while only non-augmented images were provided to the model during validation and testing. A total of four random augmentations were performed: brightness was augmented by a factor between 0.5 and 1.5, after which images were rotated between ±180 degrees, translated by a maximum of 5 voxels in the x, y and/or z axis, and finally flipped across the sagittal or axial plane or both. In order to ensure that no ‘black borders’ (i.e. areas with no image data due to rotation and shifting during augmentation) would be fed to the model, images were again cropped to a final volume of 48 × 48 × 48 mm^3^. Finally, a single axial central slice was provided to the model as input.

**Figure 4:**
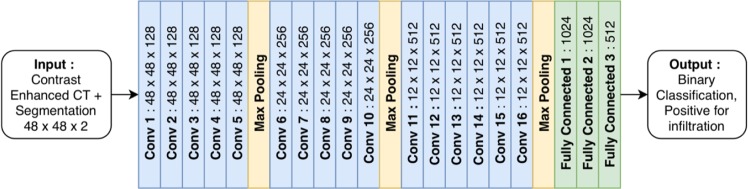
Convolutional neural network architecture. All three CNNs developed shared a common architecture and differed by the data used for training. CNNs received 2D contrast-enhanced CT images and segmentation masks as input, with input images augmented randomly during training. All convolutional layers used a kernel size of 3 × 3. A rectified linear unit (ReLU) activation function followed by batch normalization was performed at every layer. Adam optimization was used to update network weights, with parameters for alpha, beta1, beta2 and epsilon set at 0.0001, 0.9, 0.999 and 1e-08. Training was continued for 50 epochs.

Networks received two-dimensional images of 48 × 48 voxels and output a binary prediction whether or not the single lymph node displayed contained tumor or not. A final network architecture with 16 convolutional layers and three densely connected layers, inspired by the success of similar architectures by the Visual Geometry Group (VGGNet)^21^, was selected using k-fold validation with k = 10. Figure 4 shows the architecture used by all CNNs. CNNs were not pre-trained. Batch normalization was performed after every layer, with rectified linear units (ReLU) used as the activation function. The output of the convolutional layers was fed to a fully connected feed forward network with 3 hidden layers. Adam optimization was used to update network weights^22^, with parameters for alpha, beta1, beta2 and epsilon set at 0.0001, 0.9, 0.999 and 1e-08.

Three separate CNNs were trained. All models shared identical network architecture and were distinguished by the dataset used to train them: the status balanced model received status balanced CT images and segmentations, the location balanced model received location balanced CT images, and the xMask model received status balanced CT images multiplied by their corresponding segmentation mask. All models were implemented in Keras and Tensorflow (v. 1.10.1) and run on a Nvidia TITAN Xp graphics card (NVIDIA Titan Xp, Rev A1, Santa Clara, CA, United States). Heatmaps were generated using the Innvestigate (v. 1.0.2) package^23^ using the PatternAttribution method^24^.

### Random forests

In order to validate neural network performance, random forests were generated to predict nodal infiltration status taking only nodal volume in mm^3^ and nodal anatomical location into account. Two random forests were trained for each of the training sets used (status balanced, location balanced). Anatomical location was encoded as a one hot vector. Random forests were implemented using the sklearn python package^25^ with maximum depth set at 5 to prevent overfitting to the training data.

### Study readers

Two radiologists, with at least 5 years of experience in urogenital imaging, were presented with all test CT images (n = 130). Radiologists were presented an 80 × 80 × 80 mm^3^ volume centered on the lymph node in question at 1 × 1 × 1 mm^3^ resolution, and were asked to categorize the likelihood of lymph node infiltration by tumor from the following four categories: very unlikely, unlikely, likely, and very likely. Neither the segmentation, 68Ga-PSMA PET/CT images, nor label were provided.

### Statistical analysis

Model performance was evaluated for each CNN on the independent test set (n = 130) using the area under curve (AUC) of the receiver operating characteristic (ROC) curve. AUCs and confidence intervals were calculated using the pROC package in R^26^, with confidence intervals computed using the bootstrap method with 10,000 stratified replicates. To allow for model comparison, the optimal threshold at which to consider CNN output as positive was set by maximizing Youden’s index (sensitivity + specificity − 1), from which binary predictions were generated. Accuracy, sensitivity, specificity, PPV and NPV were calculated using the binary predictions. For study readers, the four categories were simplified to a dichotomous prediction of likely/unlikely. AUC for each radiologist is equivalent to the average of specificity and sensitivity^27^. McNemar’s test was applied to all pairs of CNNs and experts. Results were considered statistically significant at a reduced P < 0.005 level to correct for multiple comparison. All variables are given as mean along with standard deviation and range where applicable.

## Results

### Evaluation of CNN classifiers and experts

**Figure 5:**
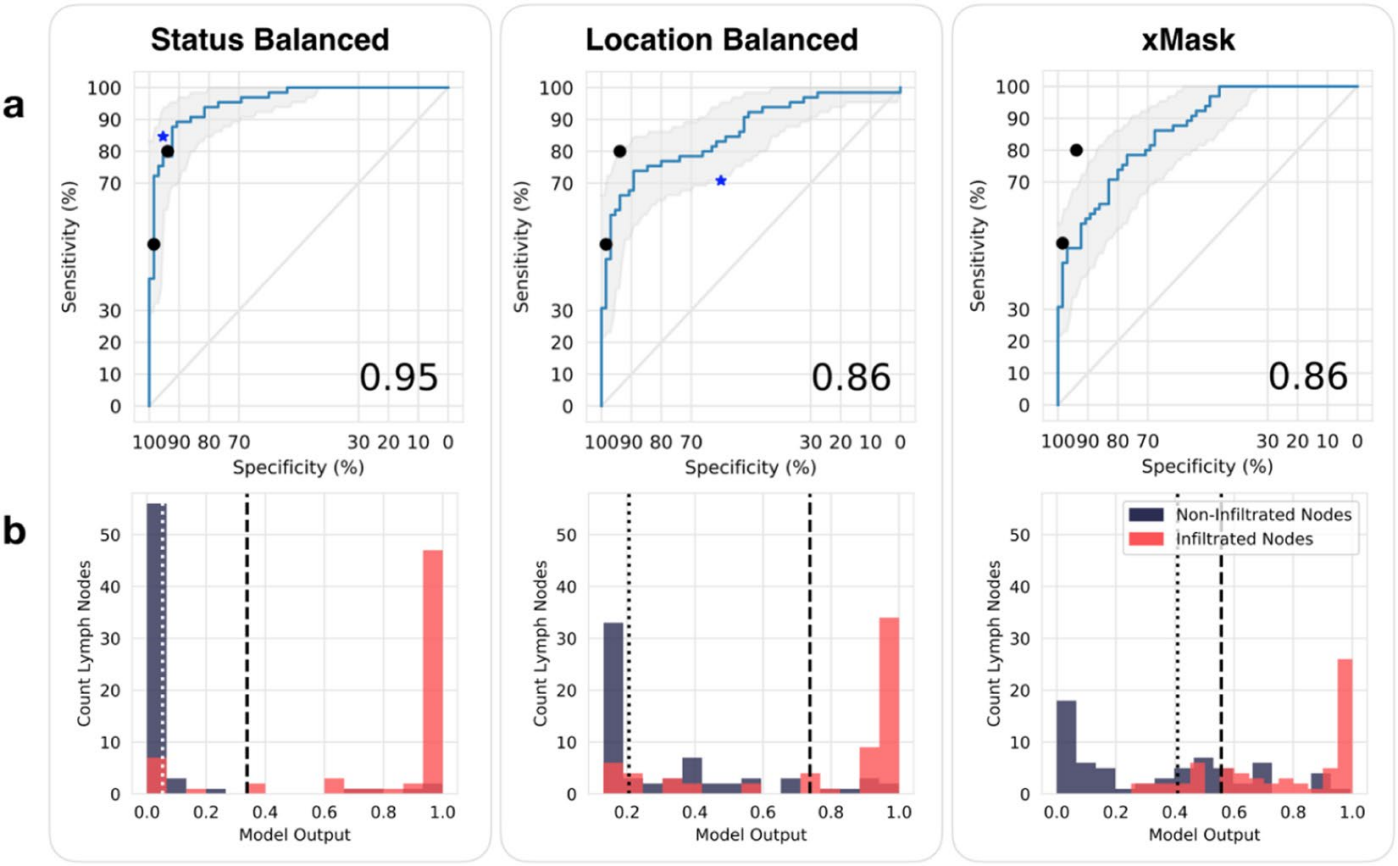
Classification performance. (**a**) Shown are the ROC curves for the three trained CNNs on the separate test set (n = 130) with 95% confidence interval of the sensitivity at given specificities in shaded gray. Displayed in the lower right hand corner is the corresponding AUC. Classification by individual radiologists on the same test set are displayed as black dots. Blue stars show random forest performance on the separate test set using the corresponding training dataset (status or location balanced). (**b**) Histograms of CNN model classification performance on the test set. The threshold that maximizes Youden’s index is shown as a dashed line. The threshold which corresponds to a 90% sensitivity is shown as a dotted line. Infiltrated nodes (red bars) to the right of the given threshold are ‘true positive’, while those to the left are ‘false negative’: non-infiltrated nodes (blue) to the left are true negative, to the right are false positive.

The best performing Neural Network was trained using the status balanced training set, with an AUC of 0.955 (95% CI from 0.923–0.987). The CNNs trained with datasets where implicit frequency data was stripped using 50:50 class balancing by location category (the location balanced training set) or masking by the segmentation masks (xMask) performed comparably well, with an AUC of 0.858 (95% CI from 0.793–0.922) and 0.863 (95% CI from 0.804–0.923), respectively. Setting the sensitivity at 90% for all CNN models, the specificities of status balanced, location balanced, and xMask models was 88%, 52%, and 55%, respectively. Figure 5a shows ROC curves of all CNNs. Figure 5b shows histograms of CNN classification performance. Table 1 presents classification performance.

**Table 1:** Classification performance.

Classifier	AUC	Accuracy (%)	Sensitivity (%)	Specificity (%)	PPV (%)	NPV (%)	F1 Score
CNN: Status Balanced	0.95	89	86	92	91	86	88
CNN: Location Balanced	0.86	80	72	89	87	76	78
CNN: xMask	0.86	76	76	76	76	76	76
RF: Status Balanced	0.90	90	84	95	94	86	89
RF: Location Balanced	0.65	65	70	60	63	67	67
Expert 1	0.86	86	80	93	92	82	85
Expert 2	0.75	74	50	98	97	66	66

Classification results are displayed in percentages. The optimal threshold for the three CNNs was selected by maximizing Youden’s Index. RF: Random Forest.

The experienced uroradiologists achieved an average AUC, sensitivity, specificity and accuracy of 0.81, 65%, 96% and 81% respectively. The first radiologist performed with a calculated AUC of 0.86, while the second radiologist achieved a calculated AUC of 0.75. All differences in error rate between CNNs and expert readers was not statistically significant using McNemar’s test and p set at a reduced 0.005.

The random forest trained with the status balanced training set achieved an AUC, sensitivity, specificity and accuracy of 0.900, 84%, 95% and 90% respectively on the test set. The random forest trained with the location balanced set performed significantly worse with an AUC, sensitivity, specificity and accuracy of 0.654, 70%, 60% and 65% respectively on the test set.

### Use of heatmaps to explain differences in performance

**Figure 6:**
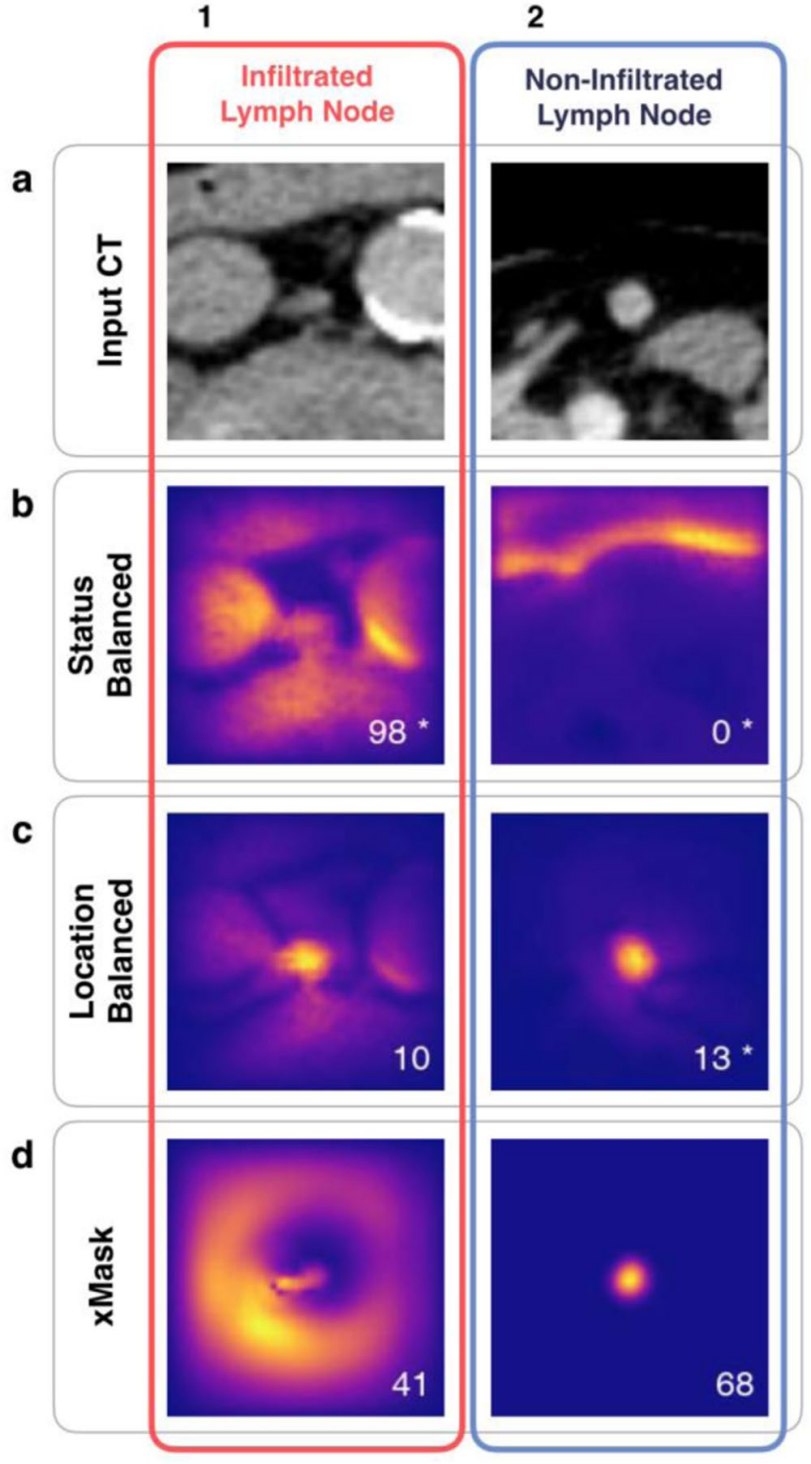
Heatmaps display neural network attention. (**a**) Contrast-enhanced CT images for two lymph nodes that were used as input to generate all heatmaps displayed, with (1) a retroperitoneal lymph node positive for infiltration by PCa, and (2) an inguinal lymph node negative for infiltration. In (**b–d**) heatmaps for the lymph nodes shown in (**a**), produced by three CNNs trained with status balanced training data, location balanced training data, or masked input data, respectively. CNN output, a pseudo probability score that the lymph node was classified as positive for tumor infiltration, is shown in the bottom right of each heatmap in (**b–d**). Stars signify true output predictions (either true positives for the lymph node in column 1 or true negative for column (2), with thresholds set by optimizing Youden’s index for each CNN, set at 34, 73 and 54 for b,c, and d respectively. Within heatmaps, light colors represent areas that contribute to output prediction, while dark regions contribute little to output prediction. CNNs often highlight regions within the lymph node that expert radiologists recognize as important for infiltration status, such as nodal center density and contrast enhancement. In true positive images it appears that high central density is the most relevant parameter in designating a ‘positive’ label. We postulate that the ‘halo’ surrounding the lymph node in the xMask CNN (d1), depicts the CNN attention to size. Heatmaps produced by the CNN trained with status balanced data highlight anatomical regions which aid in classification of lymph nodes, often demarcating the air-skin border seen in images of inguinal lymph nodes, as in b2.

**Figure 7:**
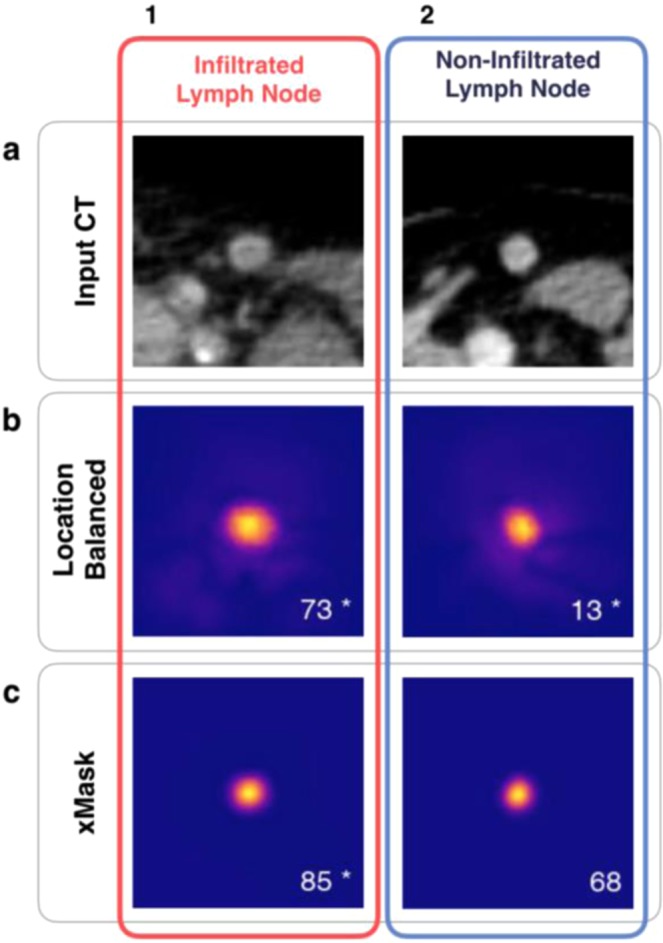
Limitations of heatmaps as tool to explain black box predictions. (**a**) Contrast-enhanced CT images for two inguinal lymph nodes that were used as input to generate all heatmaps displayed, with (1) a lymph node positive for infiltration by PCa, and (2) a lymph node negative for infiltration. In (**b,c**) heatmaps produced by two CNNs trained with location balanced training data, or masked input data, respectively. Beyond verifying that the lymph node is important for classification, heatmaps provide little additional information as to why classification output was either true positive (b1,c1), true negative (b2), or false positive (c2).

Using heatmaps, we sought to elucidate how deep learning achieves a high classification performance. Examples of heatmaps are shown in Figs. 6 and 7. It appears that the CNNs are able to learn features within the lymph node and more surprisingly, outside the boundaries of the lymph node (such as the aorta or air/skin borders), that correlate with lymph node infiltration status. It is critical to note that our best performing model, trained on status balanced data, appears to rely on features outside of the lymph node in question. This can be most clearly seen on images of inguinal or mediastinal lymph nodes, where areas of skin/air border (often found in the inguinal region) or lung/mediastinum border contribute heavily to final classification output, and the lymph node centered in the image is not highlighted. Heatmaps from the same CNN show that the lymph node itself is more important in true positive considerations, suggesting that ‘inguinality’, i.e. features of the inguinal region are important considerations in a negative infiltration status. Heatmaps generated can also be diffuse, with CNN attention displayed in many regions of the image but not particularly focused on the lymph node or surrounding region.

## Discussion

In this study we trained and tested three CNNs that predict metastatic infiltration of lymph nodes by PCa using contrast-enhanced CT images and assessed their performance versus that of experienced human readers. The CNNs performed at the same level of two expert radiologists.

Current attempts at detecting lymph node metastases in PCa by radiological reading have been shown to be suboptimal, with a sensitivity and specificity shown in one study to be 42% and 82%, respectively^6–8^. Size is often the most relevant diagnostic criteria, with nodes greater than 10 mm deemed as suspicious and all below as benign^9,10^. Other criteria are difficult to quantify and are highly dependent on reader experience. Thus, the use of quantitative or algorithmic methods to detect LNI is desired. By radiomic analysis, in which a host of quantitative features are extracted from images and analyzed for statistical correlations, it has been suggested that a 7.5–Hounsfield CT density threshold could act as a surrogate parameter to differentiate LNI from benign processes^28^, with 89% of non-infiltrated LNs below this threshold and 92% infiltrated LN above, though this study used many different cancer types and a mix of PET tracers as a standard of reference. Deep learning, in which optimization algorithms are used to train neural network models in classification tasks, have shown mixed success in detecting LNI. It has been previously found that CNNs are able to predict SUVmax in a PET scan using CT images of lymph nodes with a moderate accuracy, with an AUC of 0.85^29^. A number of studies predicting mediastinal LNI by lung cancer and breast cancer have been performed, with one study finding an AUC of 0.76^30^ and another study classifying LNI in axillary lymph nodes by breast cancer achieving an AUC of 0.84^31^. CNNs were also found to classify head and neck tumor extranodal extension with an AUC of 0.91 using 3D CT images^32^. It has also been shown feasible to identify tumor infiltrated lymph nodes in MRI using deep learning^33^. To our knowledge, no study using deep learning to identify metastases of PCa into the lymphatic system by CT has been performed so far.

Generation of heatmaps is an attempt to explain how deep learning models reach classification decisions on a per-image basis, and represents a growing field of research known as ‘explainability’. Each heatmap can be interpreted as displaying CNN attention; regions of an input image that influenced the classification decision are demarcated. From the heatmaps produced in our study, it becomes clear that identical CNN architectures learn different methods to solve the same problem, depending on which data is used for training. It appears that the CNN trained with status balanced data learned not only to recognize features of the lymph node in question, but also to recognize anatomical features surrounding the lymph node. Using these anatomical features, it appears that the status balanced CNN implicitly learned frequency of infiltration in different anatomical regions and used these frequencies or probabilities to improve output prediction. For example, in the status balanced dataset, 91% of inguinal lymph nodes were negative (see Fig. 3a). Thus, labeling all inguinal lymph nodes as negative is highly rewarded during the training process, and recognizing ‘inguinality’ aided in achieving high classification accuracy. Indeed, the air/skin border found in inguinal lymph nodes was often well demarcated in heatmaps, as seen in Fig. 6. However, it is unclear to what extent such anatomical features influenced classification; the CNN trained with status balanced data did classify some inguinal lymph nodes as positive, and some retroperitoneal lymph nodes (of which 94% were positive in the status balanced dataset) as negative, as shown in Fig. 5b. The fact that learning anatomical features within the image (as proxy for anatomical location) greatly improves classification performance in the status balanced dataset is underscored by the high performance of the random forest trained on the this dataset; using nodal volume and location alone, high classification performance was achieved (AUC 0.90). Thus, our best performing neural network is most likely essentially useless on external datasets not sharing the anatomical bias found in the status balanced dataset.

We created two additional CNNs to eliminate anatomical clues within images in an attempt to force neural network attention to the lymph node. First, we created a new training dataset created by balancing positive and negative lymph nodes within each location category. By doing so we eliminated the possibility of learning infiltration frequency at each anatomical location. While it is clear from generated heatmaps that the CNN trained with this location balanced set did focus more on the lymph node and not on anatomical features, it was not able to achieve the same classification performance as the status trained CNN. However, a random forest receiving nodal volume and location information trained on this location balanced dataset performed poorly, considerably worse than the CNN (AUC 0.677 vs 0.858). This leads us to believe that the neural network is indeed focusing on features within the lymph node to perform classification. Secondly, a new CNN was provided images created by multiplying the CT image by the manually generated segmentation (xMask), thus setting all values outside of the lymph node to zero. This removed all contextual information, such as location in the body or presence of neighboring structures. The resulting performance was similar to the location balanced CNN. Interestingly, heatmaps created by the xMask CNN often showed a diffuse halo like pattern of attention outside of the lymph borders, which we postulate may be the CNNs attention to size. We cannot definitively state that any of the CNNs developed are able to determine nodal size due to intrinsic limitations of heatmaps as an explainability tool and the black box nature of neural networks, which often created very similar looking heatmaps (see Fig. 7). Regardless, size alone is a poor predictor of infiltration, as can be intuited by the considerable overlap of volume distributions for lymph nodes positive and negative for infiltration (see Fig. 3b) and shown quantitatively by the poor performance of the random forests trained with location balanced data.

There are a number of limitations to our study. It is important to note that the usage of PSMA PET/CT is an imperfect method of label generation. In comparison to the gold standard for detecting LN metastases, namely histopathological analysis after extended pelvic lymph node dissection (PLND)^34^, PSMA PET/CT was found to have a sensitivity of 80% and specificity of 97% in a systematic review and meta-analysis^15,16,35,36^. Due to the high specificity, it is unlikely that our models were trained with large numbers of false positive lymph nodes. In addition, we relied on manual detection of segmentation of lymph nodes, and we do not perform lymph node detection. The tendency to select easily definable and large lymph nodes for analysis led to a large amount of inguinal lymph nodes being included in our dataset, a limitation we sought to overcome by various means of class balancing.

The obvious attention to anatomical features demonstrated by our best performing CNN raises a number of issues in the implementation of deep learning in the medical field. Deep learning models are able to learn frequencies and summary statistics, known as biases, within datasets, which can lead to high classification performance based upon undesirable features. This problem is distinct from overfitting to the training dataset, and instead points to the need for a more rigorous explainability of deep learning models. Our results represent a moderate success in the use of saliency maps (heatmaps), as through this instance-based analysis of CNN attention, we were able to determine that our best performing model was using anatomical features of the lymph node environment in addition to features within the lymph node. We were able to compensate for anatomical variations in infiltration frequency because we had collected coarse data on anatomical location. However, not only does class balancing at ever higher levels of abstraction encroach on the notion of ‘automated feature generation’, it is not feasible in the medical field due to lack of knowledge of what constitutes a relevant category. The lack of explainability methods for deep learning models is also a limitation. Our use of heatmaps, known as an attribution method, of which there are several, is problematic not just because of inconsistencies in implementation and performance^24^, but the underpinning assumption that individual pixels in an input image should be the primary unit of relevance for classification.

Current deep learning systems can perform remarkably well and will most likely continue to improve with larger datasets and access to more contextual information, such as blood serum values and genomic data. Our results show that CNNs are capable of classifying lymphatic infiltration by PCa on contrast-enhanced CT scans alone as compared to the 68Ga-PSMA PET/CT reference standard. Anatomical context influences the performance of CNNs and should be carefully considered when building such imaging based biomarkers.
